# Florida Harvester Ant Nest Architecture, Nest Relocation and Soil Carbon Dioxide Gradients

**DOI:** 10.1371/journal.pone.0059911

**Published:** 2013-03-28

**Authors:** Walter R. Tschinkel

**Affiliations:** Department of Biological Science, Florida State University, Tallahassee, Florida, United States of America; Arizona State University, United States of America

## Abstract

Colonies of the Florida harvester ant, *Pogonomyrmex badius*, excavate species-typical subterranean nests up the 3 m deep with characteristic vertical distribution of chamber area/shape, spacing between levels and vertical arrangement of the ants by age and brood stage. Colonies excavate and occupy a new nest about once a year, and doing so requires that they have information about the depth below ground. Careful excavation and mapping of vacated and new nests revealed that there was no significant difference between the old and new nests in any measure of nest size, shape or arrangement. Colonies essentially built a replicate of the just-vacated nest (although details differed), and they did so in less than a week. The reason for nest relocation is not apparent. Tschinkel noted that the vertical distribution of chamber area, worker age and brood type was strongly correlated to the soil carbon dioxide gradient, and proposed that this gradient serves as a template for nest excavation and vertical distribution. To test this hypothesis, the carbon dioxide gradient of colonies that were just beginning to excavate a new nest was eliminated by boring 6 vent holes around the forming nest, allowing the soil CO_2_ to diffuse into the atmosphere and eliminating the gradient. Sadly, neither the nest architecture nor the vertical ant distribution of vented nests differed from either unvented control or from their own vacated nest. In a stronger test, workers excavated a new nest under a reversed carbon dioxide gradient (high concentration near the surface, low below). Even under these conditions, the new and old nests did not differ significantly, showing that the soil carbon dioxide gradient does not serve as a template for nest construction or vertical worker distribution. The possible importance of soil CO_2_ gradients for soil-dwelling animals is discussed.

## Introduction

Many ant species excavate nests in the soil, creating species-typical architectures of a wide range of sizes and shapes [1]–[9] (more complete reviews can be found in the papers of Tschinkel and coworkers). Most subterranean ant nests are composed of modular units consisting of horizontal chambers connected by more-or-less vertical shafts [3]. Species-typical architectures evolved through differences in the size, arrangement, spacing and shape of any or all of these elements, and also by combining multiple chamber-shaft units into more complex nests. The ants enlarge the nest as the colony within grows, but all elements are enlarged simultaneously so that the overall size-free shape of the nest does not change, and the species-typical appearance is retained [1]–[4], [8], [9].

Because ants build their nests in the dark without a leader or blueprint and the nest is the product of group effort, ant nests would appear to be preeminent products of self-organization. The central question is what behavioral programs, social interactions, environmental cues and interactions with the forming nest direct the ants to create these species-typical nests? One approach to this question has been through laboratory experiments in which ants excavate sand from the spaces between glass plates [10], [11] or small, soil-filled containers [12], [13]. The resulting chambers are analyzed mathematically for patterns. However, whether or not the results resemble real nests in nature is an open question.

To some degree, the vertically patterned structure of the nests themselves, as revealed by making casts [14], suggests possible environmental cues that the ants might use to guide their efforts. Thus, Tschinkel [1] noted the striking correspondence between the soil CO_2_ gradients and the top-heavy architecture of subterranean harvester ant nests (*Pogonomyrmex badius*), as well as the distribution of the ants within these nests, and proposed that the CO_2_ gradient could serve as the template that guided the ants in the creation of these vertical patterns. This hypothesis was made more plausible by the fact that many insects can smell CO_2_ [15, see also 16 for examples], and that certain neurons in the insect central nervous system are specifically CO_2_ sensitive. Moreover, ants have been shown to vary their digging behavior in response to CO_2_ [17]. Leafcutter ants have specialized receptors that respond to CO_2_ but do not habituate, thus allowing the accurate assessment of CO_2_ concentrations, which in turn regulate ventilatory behavior [18]–[21]. The genes encoding specific CO_2_ receptors in *Drosophila melanogaster* have been identified with paralogs in several other (but not all) species of insects [16].

Soil, the medium in which ants dig their nests, plays a major role in the world’s carbon cycle by releasing the carbon dioxide produced by root and microbial respiration. This efflux of CO_2_ from the soil surface back to the atmosphere has been the frequent subject of climate-system studies [22], and many estimates of its rate, relationship to ecotype, temperature, soil moisture and other physical factors have been made [for example, 23]. Because the efflux of CO_2_ to the atmosphere occurs only at the soil surface, most soil CO_2_ measurements have been made no more than a few cm below ground. However, a small number of studies have shown that soil CO_2_ concentration increases with depth, sometimes by one to two orders of magnitude, and typically produces a non-linear gradient [24]–[28]. A summary of the physics of such gradients can be found in Holmes [29], who notes that the combined effects of CO_2_ production, soil moisture and efflux to the atmosphere should produce a gradient that reaches maximum concentration at the water table.

Whereas the nature and behavior of such gradients has been shown to affect surface efflux of CO_2_, their effects on soil-dwelling creatures has rarely been studied. Because the atmospheric pressure in soil pore spaces differs little from the general atmospheric pressure, higher CO_2_ partial pressures must be accompanied by lower O_2_ partial pressures [27]. Organisms living in soil must therefore deal with both elevated CO_2_ and reduced O_2_, both of which have important effects on the physiology of a wide range of animals, including mammals and insects [30]. At very high concentrations, CO_2_ is toxic, and is commonly used as an anesthetic for insects. Thousands of people and livestock died of asphyxia when Lake Nyos (Cameroon) emitted catastrophic amounts of CO_2_ in 1986. For animals that burrow deeply in the soil, elevated CO_2_ and reduced O_2_ concentrations present a physiological and behavioral challenge that must be met if the animal is to flourish. Even fungi and other microbes are affected by elevated soil CO_2_ concentrations [31]. High CO2 concentrations negatively affect the symbiotic fungus cultivated by leafcutter ants [32].

Aside from the importance of the absolute concentration of CO_2_, the strong and regular increase of CO_2_ concentration with depth could potentially provide animals with a proxy for depth, allowing depth-appropriate responses. Among these would be depth-structured burrows and subterranean nests such as those excavated by ants and other ground-nesting insects. On the basis of theoretical calculations, Cox and Blanchard [33] proposed that the carbon dioxide gradients produced by ants nesting between stones could provide a template for wall construction and social arrangement, but their study did not consider soils, nor did it test actual gradients in nests. However, Scholes et al. [34] found no evidence that the ants use such gradients when sorting brood.

The purpose of this paper is to describe north Florida soil CO_2_ gradients, to show that they contain reliable depth information and to determine how this information varies in space and time. Most importantly, I test whether these soil CO_2_ gradients are actually used as a depth-template by Florida harvester ants during the excavation of their nests.

## Materials and Methods

### Ethical Statement

This study was carried out in the Apalachicola National Forest under US Forest Service permits APA583 and APA56302. No protected species were involved. Excavations were refilled and their sites were generally difficult to detect after about a year. Colonies collected alive were returned after census and allowed to re-establish nests in their own territories.

### Study Site

The study population of Florida harvester ant, *Pogonomyrmex badius*, is located in a 23 ha site (latitude 30.3587, longitude −84.4177) about 16 km southwest of Tallahassee, Florida, USA, within the sandhills portion of the Apalachicola National Forest. The site, Ant Heaven, consists of excessively drained sandy soil occupying a slope to a wetland and stream, causing its water table to be depressed (>5 m at the maximum), thereby making it suitable for *P. badius* and *Solenopsis geminata*, as well as several drought-resistant species of plants such as *Opuntia* and *Nolina*. The area also supports a population of gopher tortoise (*Gopherus polyphemus*). The forest consisted of longleaf pines (*Pinus palustris*) planted ca. 1975, turkey oak (*Quercus laevis*), bluejack oak (*Quercus incana* ) and occasional sand pines (*Pinus clausa*) and sand live oak (*Quercus geminata*). Because the soil had been disturbed in the early 1970s, the natural ground cover of wiregrass (*Aristada stricta*) was absent, replaced by broomsedge (*Andropogon* spp.) and several other successional species. The same disturbance may have helped establish this dense population of *P. badius,* whose nests are easily spotted because the ants decorate the excavated soil disc with a layer of charcoal bits (mostly the ends of burned pine needles) [35]. The black charcoal contrasts sharply with the light-colored sand or litter.

### Population Mapping

This study required the detection of early stages of colony relocation. To facilitate this, each nest was marked with a vinyl flag and numbered metal tag, and its location recorded on a Trimble GeoExplorer CE mapping GPS instrument. Location data were differentially corrected using the base station maintained by the Department of Environmental Protection in Tallahassee, resulting in a final precision of approximately 50 cm. The population was resurveyed several times between March and December in order to map completed or current relocations and newly detected colonies. The tracked population numbered about 350 colonies.

### Excavation and Mapping of Nests

Excavation and chamber mapping methods were similar to those of Tschinkel [36]. A pit large enough to work in was dug next to the focal nest, and the chambers exposed one by one by lifting off the soil in horizontal layers to expose the outlines of each chamber. A battery-powered shop vacuum was used to clear remaining sand from chambers before a sheet of clear acetate was laid over the chamber and its outline traced. The tracing was located in an x,y,z grid by noting its coordinates in cm in reference to a 0, 0, 0 point on the ground surface. All nest contents were collected by aspirator or vacuum and later counted in the laboratory. Together, these data allowed the 3-dimensional reconstruction of the nest chamber arrangement and nest contents as in Tschinkel [1].

### Soil Carbon Dioxide Gradients

Initially, gas samples were withdrawn from various depths of living and abandoned harvester ant colonies with a 2 m long “syringe needle”, that is, a very fine (2 mm o.d.) stainless steel tube provided with a point at the lower end and a Luerlok syringe fitting at the upper end. The fine tube was encased in a stouter (4.5 mm diam.) steel tube that could be pushed into the soil to specified depths. A 60 ml plastic syringe was then used to withdraw a gas sample, and this sample was injected into a portable CEA Instruments Carbon Dioxide Model 888 Analyzer to estimate the concentration of CO_2_. Raw readings were corrected by injecting a standard CO_2_-air mixture every 5 readings.

**Figure 1:**
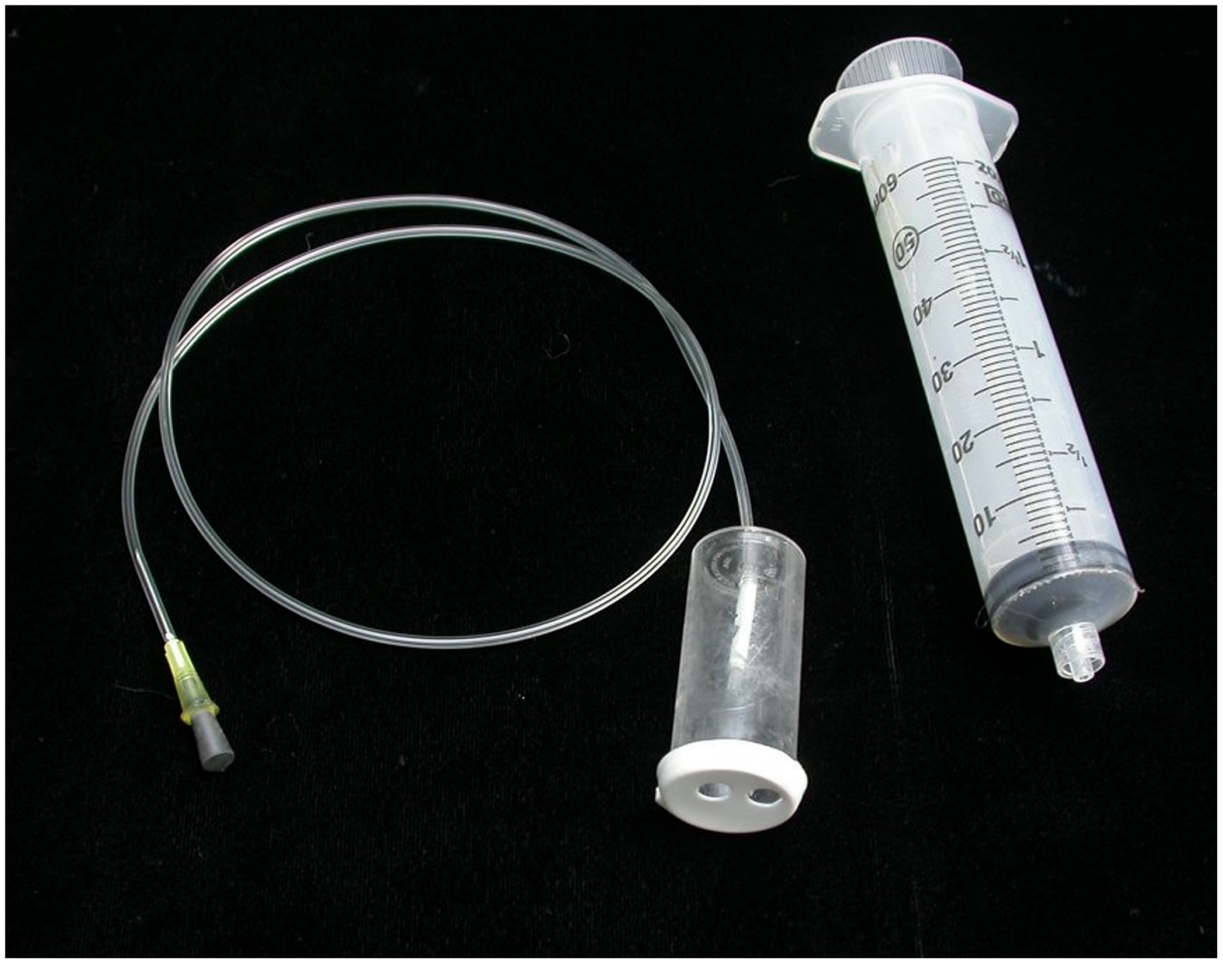
Equipment used to sample soil gas at several depths. The vial was buried openings-down with the fine tubing and syringe needle extending to just above the soil surface. A syringe-full of gas could then be withdrawn from the vial after removing the stopper from the needle. CO_2_ in the sample was measure by injecting it into a portable CO_2_ analyzer. Four such vials were planted in each borehole at 4 different depths (15, 35, 75, 175 cm).

Once it became apparent that the CO_2_ gradients were not created by the ants, but were a property of the soil, permanent sampling arrays were established at 12 locations throughout the site such that the high-to-low profile of the topography was transected at three locations. The basic sampler consisted of a 5 dram polystyrene vial with a fine vinyl tube piercing the bottom of the vial and opening into the air space within the vial. The vial cap had two holes punched into it, and a 22 ga syringe needle was inserted into the other end of the thin vinyl tube (Fig. 1). These units were constructed with different lengths of vinyl tubing (15, 35, 75, 175 cm made up a set), and these sets were buried in a bore hole such that each sample vial opened to the soil at the specified depths. Samples were withdrawn from each depth for analysis by connecting a 60 ml syringe to the needle at the ground surface, and the sample was injected into the CO_2_ analyzer. The arrays were sampled several times between May 2011 and January 2012.

Such arrays were also established on transects that had previously been used to study the relationship of ant communities to the depth of the water table [37]. In contrast to the sandhills site of the main *P. badius* population (“Ant Heaven”), these transects were all located in the flatwoods region where the water table ranged from 0 to 2 m below the ground surface, facilitating the correlation of groundwater and CO_2_ gradients.

### Manipulation of the Soil CO_2_ Gradient

**Figure 2:**
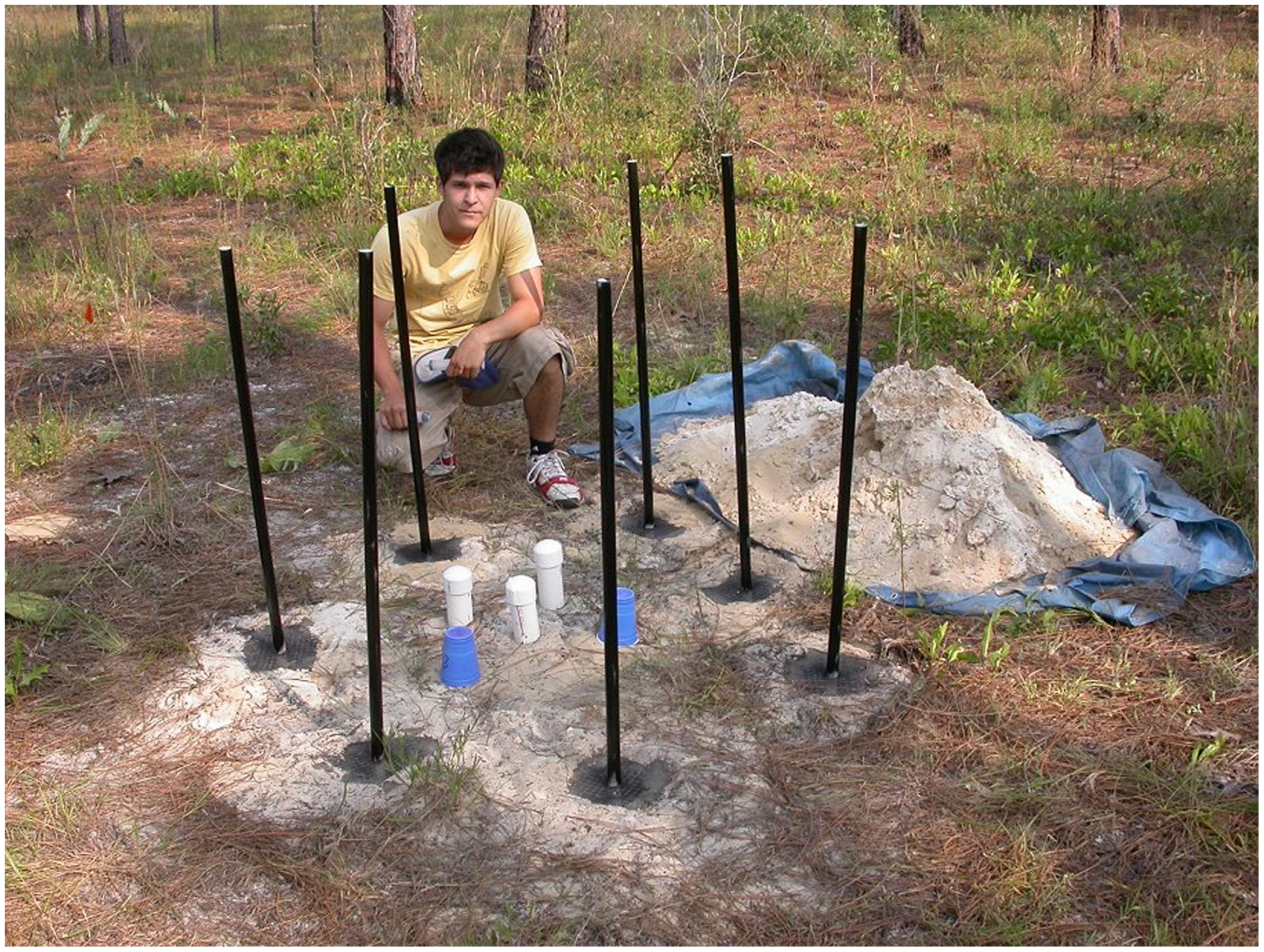
The arrangement used to eliminate the CO_2_ gradient from a central cylinder of soil. The blackened PVC pipes are set in the bottom of 10 cm by 2 m deep boreholes and act as chimneys ventilating the boreholes. CO_2_ diffuses into the boreholes from the soil and is vented to the atmosphere. In this test arrangement, CO_2_ concentrations in the central cylinder were monitored by 5 sampling arrays (the syringe needles were under the short, capped PVC pipes and blue plastic cups). In this situation, the gradient disappeared in less than a day. The person in the photo has given written informed consent, as outlined in the PLOS consent form, to publication of his photograph.

The role of the CO_2_ gradient in organizing nest structure and ant distribution could be tested only upon altering the gradient to suit experimental needs. Preliminary tests showed that placing six 10 cm by 2 m deep boreholes in a circle centered on a sampling array eliminated the CO_2_ gradient. The rate of elimination was increased by placing a 3 m long PVC pipe open at both ends into each borehole, and painting the projecting pipe black to heat in the sun so that it would act as a chimney to draw out air from the bottom of the borehole (Fig. 2). A screen over the opening of the borehole prevented animals from falling in. This arrangement eliminated the CO_2_ gradient within a few hours. Filling the boreholes caused the gradient to reappear.

**Figure 3:**
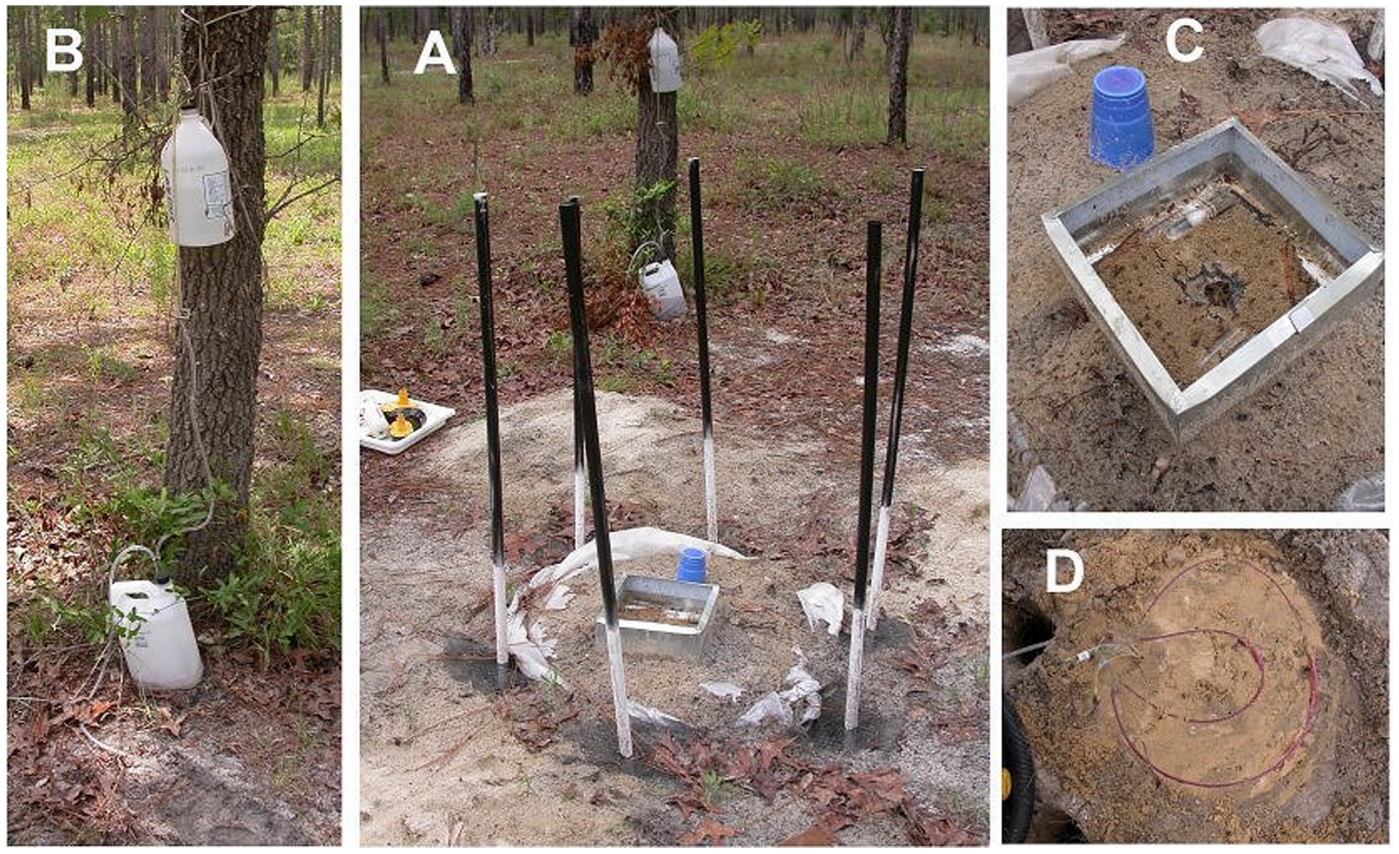
The gradient reversal setup. A. The vertical PVC pipes set in boreholes act as chimney venting the gases from the bore hole and eliminating the soil CO_2_ gradient from the central cylinder of soil. B. CO_2_ generated from HCl and lime rock was conducted to the central cylinder via tubing. C. The screen bottom cage into which the experimental colony was released. The blue plastic cup covers the syringe needles of the sampling array. D. The CO_2_ was released from perforated tubing just under the plastic sheet visible in A. Note the fine tubing and syringe needles through which gas was withdrawn from samplers at 4 depths.

A stronger test of the effects of CO_2_ gradients would be to reverse the gradient so that low concentrations occur at depth, and high just below the surface. This was achieved by first eliminating the gradient with boreholes (as above), and then piping in a constant slow stream of carbon dioxide just below the soil surface under a plastic sheet. The CO_2_ was generated through a slow drip of 0.5 N HCl onto a container of lime rock, conducting the generated CO_2_ through vinyl tubing and releasing it in the experimental setup through a ring of perforated tubing (Fig. 3). Each setup was also supplied with a sampling array so that the gradient actually generated could be measured. With careful adjustment of the HCl drip rate, this arrangement caused the CO_2_ gradient to be almost perfectly reversed.

### Subjecting *P. badius* Colonies to Altered CO_2_ Gradients

*P. badius* colonies relocate on average once a year, creating the new nest and moving into it in 4–7 days. The new nest was located between <1 m to about 10 m (one was 40 m) from the old, with a mean distance of 4 m (unpublished data). Placing six 2 m deep boreholes around nests in the initial stage of excavation caused the nest to be constructed in a gradient-free column of soil. Once the construction and move were complete, the old, vacant nest was excavated and mapped, and soon thereafter, the new, occupied nest was as well, and all contents were collected and mapped.

For the reversed-gradient experiments, colonies were collected by excavation and placed into a screen-bottom cage on top of the experimental setup, so that they had no choice but to excavate a new nest in the reversed-gradient soil through a hole in the screen bottom and plastic sheet (Fig. 3C). These nests were excavated and mapped after 10–14 days.

### Data Analysis

Data were analyzed by regression, ANOVA and t-test using Statistica 6.0. Data were log-transformed as needed to stabilize the variance.

## Results

### Carbon Dioxide Gradients in Harvester Ant Nests and in Non-nest Soil

**Figure 4:**
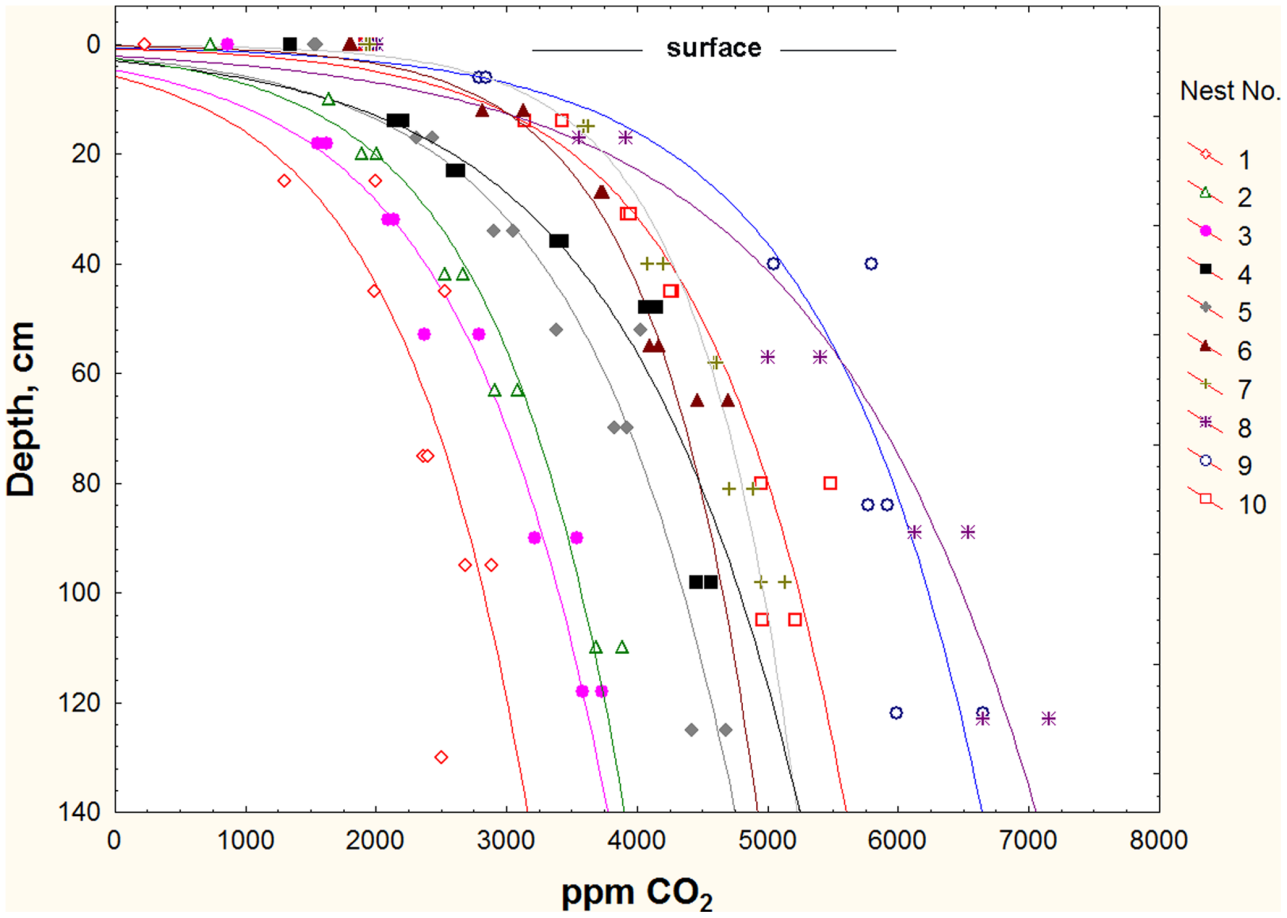
Carbon dioxide concentration in harvester ant nests in relation to depth below the ground surface. Each curve represents a different nest, some of which were active and some abandoned (ghosts). Whereas nests differed significantly, ghosts and active nests did not. The CO_2_ concentration was a linear function of the logarithm of the depth.

Fig. 4 shows the CO_2_ concentration in relation to chamber depth in 10 *P. badius* nests chosen randomly from the Ant Heaven population (another 9 are not shown to simplify the graph). In all cases, the CO_2_ concentration increased linearly with the log of the depth, that is, the gradient was logarithmic. The slopes of the log regressions for the individual nests differed significantly, ranging from about 1500 to 4400 (R^2^ exceeded 92% in all but one regression), but this relationship did not differ between active nests and abandoned ones (ghosts) (slopes: t_17_ = 0.462; n.s.; intercepts: t_17_ = 0.517; n.s.). The similarity of the gradients of active nests and ghosts suggested that the CO_2_ gradients were generated by a soil process rather than by the ants. Characteristics of the gradients are explored further below, after first testing whether the ants use the depth cues provided by the gradients.

### Soil Carbon Dioxide Gradients and *P. badius* Nest Architecture

**Figure 5:**
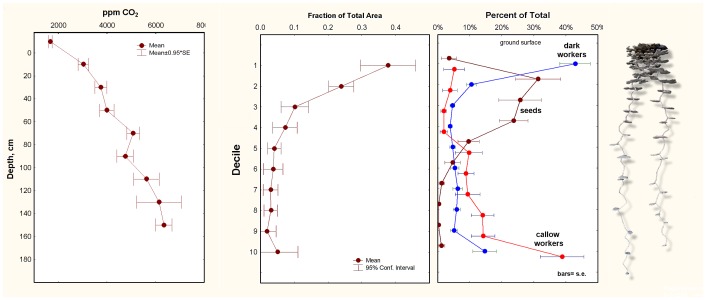
Soil CO_2_ gradients as a template for nest excavation and vertical worker sorting by age and brood stage. The ants excavate nests such that the proportion of the total chamber area at each level is inversely related to the CO_2_ concentration (Panel A and B). C. Workers and brood are vertically arranged such that the youngest (callows) and the brood are associated with high concentrations of CO_2_ and the oldest (dark workers) with low. An image of an aluminum cast of a harvester nest is shown at the extreme right.

Tschinkel (2004) suggested that the semi-logarithmic increase of CO_2_ concentrations in soil could serve as a reliable indicator of depth below the ground surface, and could therefore serve as a template for both nest construction and vertical sorting of workers by age within the nest. This hypothesis was supported by the strong correlation of the vertical distribution of chamber area in *P. badius* nests, as well as the vertical arrangement of workers by age within the nest (Fig. 5). The ability of insects to smell carbon dioxide added to the plausibility of this mechanism. As strong as this correlation and hypothesis seemed, it needed to be tested experimentally by manipulating soil carbon dioxide concentrations, as described below.

**Figure 6:**
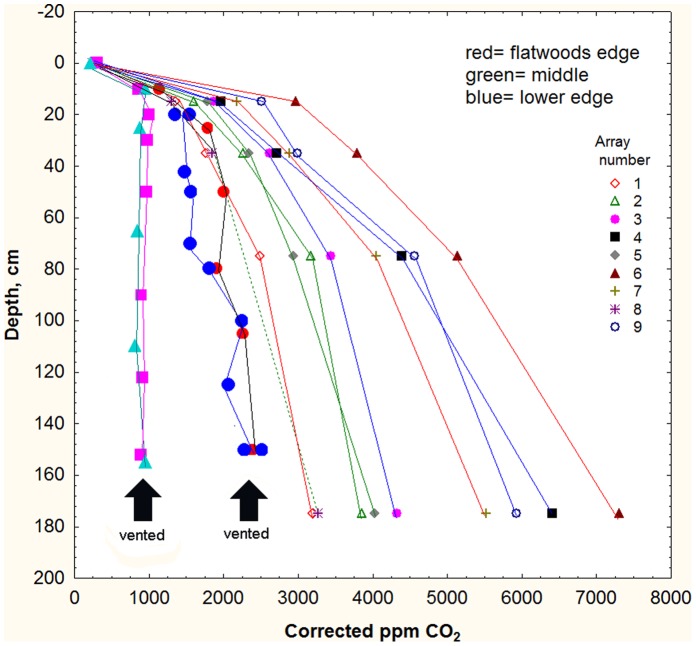
The effects of soil venting: boreholes around the perimeter of a 1 m diameter soil cylinder eliminated the CO_2_ gradient within the cylinder. Four vented trials are shown in comparison to concentrations measure in the Ant Heaven arrays.

In the first test, colonies were induced to excavate a new nest in the absence of a soil CO_2_ gradient. In the experiment, colonies that were just beginning the construction of a new nest were located, and six boreholes were placed around the perimeter of a 1 m diameter circle with the new nest at the center (see methods, Fig. 2, Fig. 6). Except for the small initial nest, most of the new nest was thus constructed in soil lacking a CO_2_ gradient. When the excavation was complete and the colony had moved into the new nest (4–6 days), the old, empty nest was excavated and mapped, and a few days later, the new nest was similarly excavated, mapped and its contents collected by level. As a control, the old and new nests of moved but unvented colonies were similarly excavated, mapped and collected. This procedure was replicated 5 times each for vented and controls. Data were analyzed by converting all to size-free measures of distribution and shape, and plotting these against 10% increments of maximum nest depth [1].

**Figure 7A–D:**
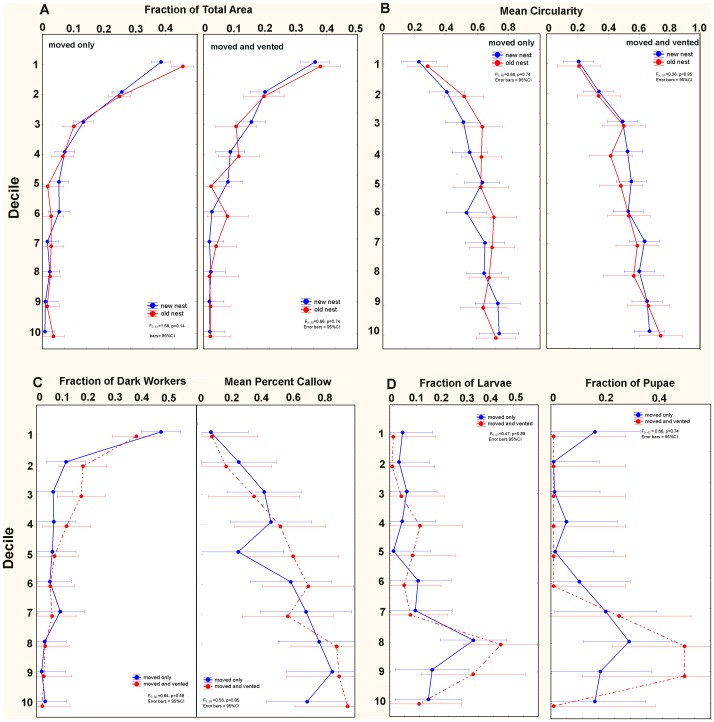
D. Size-free vertical distribution of chamber area, chamber shape, worker and brood distribution. In no measure of nest shape or ant distribution did the old and new nests of vented colonies differ, nor did they differ from the unvented nests (moved only).

In no measure of chamber area distribution, chamber shape, distribution of worker age, or distribution of brood was there a difference among the old and new nests of vented colonies or unvented (control) colonies (Fig. 7A–D). Moreover, there were no differences between new vented and new unvented (moved only) colonies in any of these measures. In all cases, chamber area distribution was top-heavy, the deeper the chamber the simpler and less lobed was the shape, dark workers were more frequent in the upper regions and callow and brood in the lower regions. These results suggested that the ants were not cueing to the soil CO_2_ gradient during nest construction or the move into the nest.

Aside from the issue of a CO_2_ template, it is perhaps peculiar that during relocation, the ants build a nest that is not only similar in size-free shape, as in Fig. 7AB, but also in absolute size. While the ants continue to enlarge the nest as the season progresses and the colony grows, it is clear that the move is not stimulated by the need for a larger nest. In spite of the high frequency of relocations, the reasons why colonies move are obscure.

**Figure 8:**
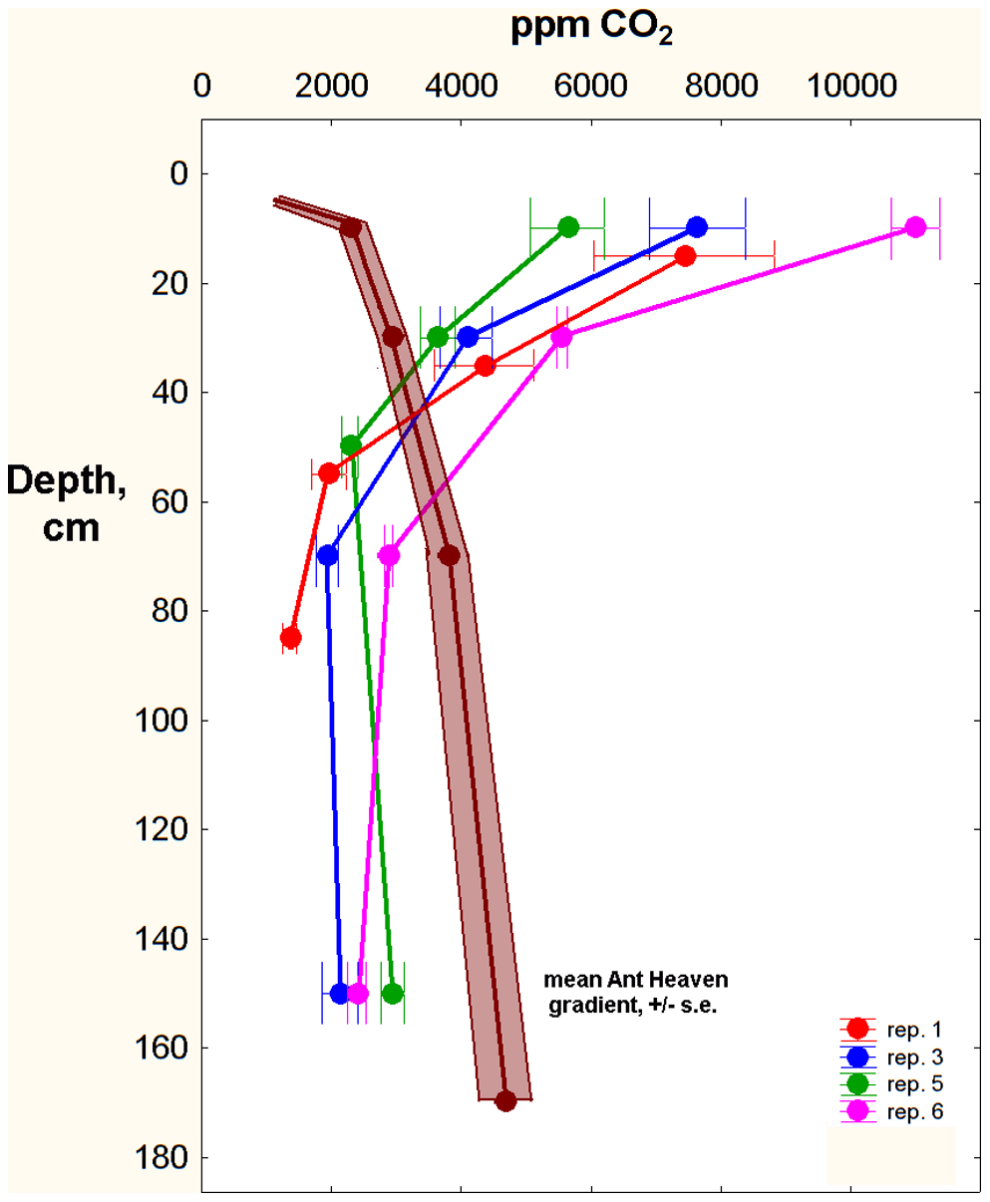
Venting plus injection of CO_2_ at the surface reversed the normal soil gradient such that CO_2_ concentrations were highest at the surface and lowest at depth (replicates 1, 3, 5, 6 in color, error bars = s.e.), the opposite of the natural gradient at Ant Heaven (mean +/− s.e. in brown). Means for the replicates were calculated from multiple readings over the duration of each replicate.

One could argue that the venting did not always completely eliminate the soil CO_2_ gradient (Fig. 6), and that the ants were able to detect very slight gradients. A stronger test would thus be to have the ants excavate a nest in a reversed soil CO_2_ gradient. Such gradients were created by first venting a cylinder of soil (as above), then piping in a slow flow of carbon dioxide just beneath the soil surface under a sheet of plastic and monitoring the actual gradient generated (see methods, Fig. 3). By regulating the HCl drip rate, it was possible to generate gradients that were almost the perfect reverse of natural gradients: 6000–10,000 ppm just beneath the surface declining to 1000–2000 ppm at 1.75 m (Fig. 8).

**Figure 9:**
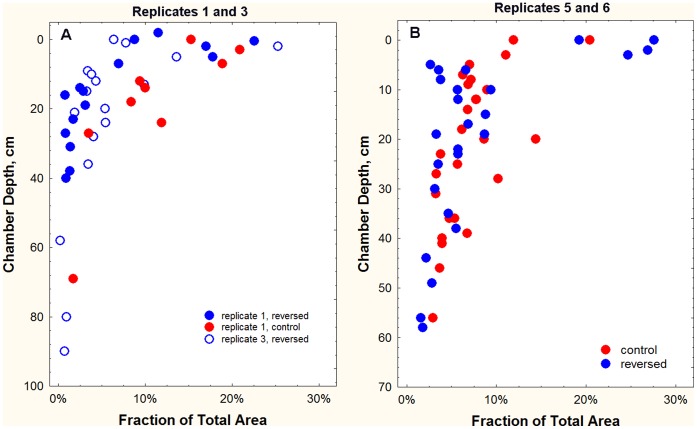
Nests excavated in reversed carbon dioxide gradients did not differ significantly from those in normal gradients (controls). A. In replicates 1 and 3, source colonies were divided into halves, one of which was planted over a normal gradient and one over a reversed gradient. The control of replicate 3 was killed by fire ants. B. In replicates 5 and 6, source colonies were mapped during excavation and then planted over a reversed gradient. The original nest thus served as the control.

Four source colonies were excavated and mapped, and then planted in screen-bottom cages over reversed gradients. All successfully excavated a nest through a hole in the screen bottom and the plastic sheet. CO_2_ gradients were measured every day or two, and colonies fed *ad libitum*. In replicates 1 and 3 (replicate 3 control was killed by *Solenopsis geminata,* as were both treatments of replicate 2), each source colony was divided into queenless halves, one planted in a control gradient, the other in a reversed gradient (Fig. 9A). In replicates 5 and 6, the source colony was mapped during excavation, and the entire colony planted in a reversed gradient. In this case, the original nest served as the control (Fig. 9B). After 9–10 days, each nest was excavated and mapped and compared to the source nest.

Reversal of the gradient had no significant effect on either the nest architecture (Fig. 9) or the distribution of ants within the nest– light colored workers predominated at depth and dark near the surface. The vertical distribution of chamber area and chamber shape was not significantly different between the source nest and the reversed-gradient nest. In spite of the difference between control and reversed in Fig. 9A, distribution of nest area was top-heavy in all treatments. Therefore, there is no evidence that the ants respond to soil CO_2_ gradients either in excavating their nests, or in arranging themselves in the vertical space within it. Sadly, a fine and appealing hypothesis has gone west.

### Factors Affecting Soil CO_2_ Gradients

**Figure 10:**
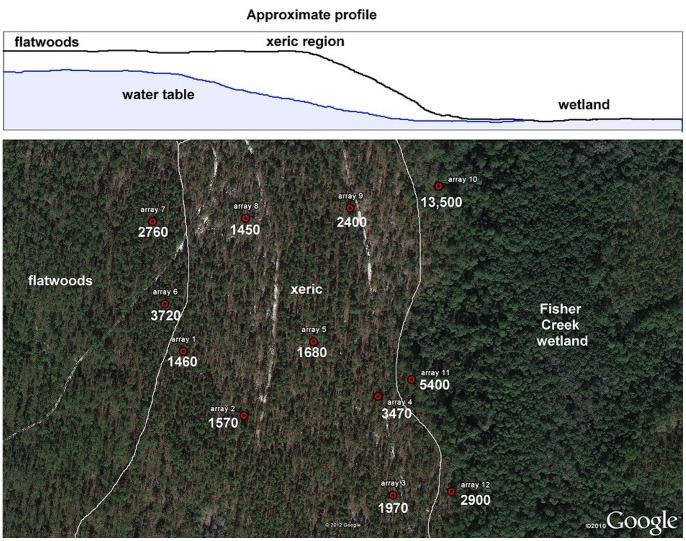
The Ant Heaven site showing the location of CO_2_ sampling arrays, the flatwoods margin and the Fisher Creek wetland. Numbers associated with each array are the slopes of the regression of ppm CO_2_ against the log of the depth, and can be interpreted as the increase in ppm CO_2_ for each ten-fold increase in depth. Maximum sampled depth was 175 cm, but the deepest sampling vials in arrays 10 and 11 were sometimes below the water table, i.e. flooded. The presence of the low-lying wetland depresses the water table under the central part of the site. Water table depth was usually <2 m at both the wetland and flatwoods margins, but >5 m in the transitional zone.

Although *P. badius* workers do not use the depth cues provided by soil CO_2_ gradients during nest excavation or vertical sorting, the potential importance of both the gradients and the elevated CO_2_ concentration in other contexts and to other creatures suggested that further exploration of their characteristics and variation would be of interest. Twelve CO_2_ sampling arrays were installed at Ant Heaven, three at the flatwoods margin, three at the Fisher Creek wetland margin, and the rest in the drier, deep sand central area (Fig. 10). These were sampled four times between May 2011 and January 2012. In all arrays, CO_2_ concentration increased with depth, much like that in the *P. badius* nests, consistent with the soil-processes origin of the CO_2_. Concentrations increased more rapidly with depth in the arrays at the flatwoods and wetland margins (Fig. 10), suggesting that a shallow water table resulted in a steeper CO_2_ gradient. Indeed, the deepest sampler in array 10 was flooded.

**Figure 11:**
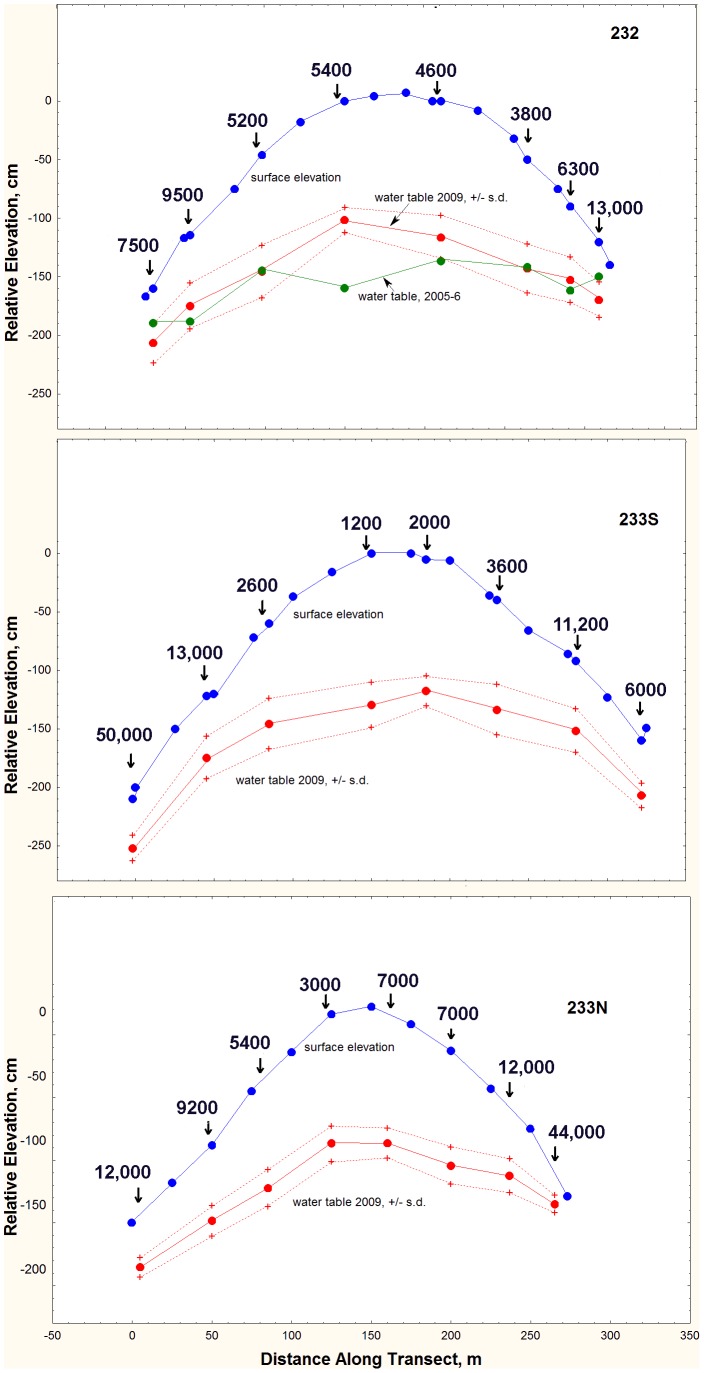
Soil carbon dioxide gradients in the flatwoods: the soil CO_2_ gradient increased in steepness as the water table became shallower. The surface elevation relative to the highest point along transects is shown in blue and elevation of the water table in red. Vertical arrows indicate the location of test wells and CO_2_ sampling arrays. Numbers over each of these locations is the slope of the regression of ppm CO_2_ vs. log depth, and can be interpreted as the increase in CO_2_ concentration for every 10-fold increase in depth.

The relationship of the CO_2_ gradient to the water table was explored further with sample arrays at the three flatwoods sites used by Tschinkel et al. [37] to determine the relationship of ant community composition to the depth of the water table. The 8 arrays and 8 test wells were arranged in a transect that ran from a wetland margin over the “summit” area about 2 m higher and then to the next wetland margin. Arrays and test wells were spaced to create roughly equal increments of depth to water. Samples were taken in June 2011 and January 2012. The surface elevation, water table elevation and steepness of the soil CO_2_ gradient are shown in Fig. 11.

**Figure 12:**
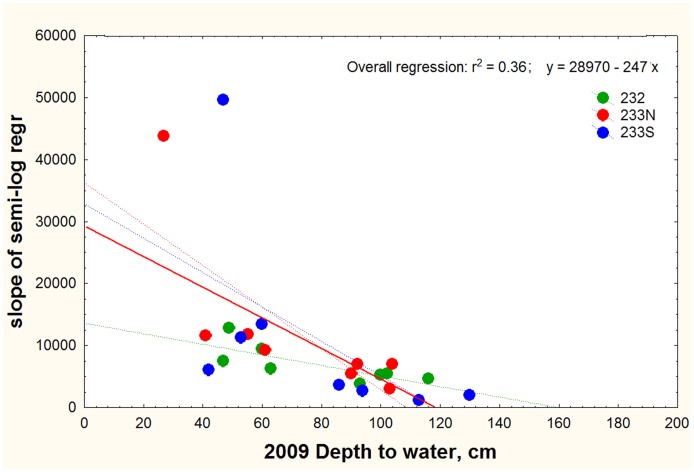
At all three flatwoods sites, the steepness of the soil CO_2_ gradient increased as the depth to the water table decreased. Note the two extremes with between 4 and 5% CO_2_, both of which were immediately above water in 2011.

The flatwoods gradients were generally steeper than those at Ant Heaven, implicating the shallow water table typical of the flatwoods (Fig. 12). Several of the deeper sampling vials at the lower ends of the transects were flooded, and samples just above the water table registered extreme CO_2_ concentrations of 4 to 5%.

To determine possible seasonal changes, the gradients at Ant Heaven were sampled four times between May 2011 and January 2012. Across these dates, the values for a given depth varied by 1700 to 2300 ppm (maximum – minimum), but this variation was not related to depth (one-way ANOVA: F_3,44_ = 3.16; n.s.). However, because CO_2_ concentrations increased with depth, the coefficient of variation (COV) decreased from about 28% at 15 cm to 16% at 175 cm. The highest concentrations at all depths occurred in late June, 2011, perhaps as a result of rainfall or temperature.

Soil type also affected the CO_2_ gradient. Two arrays in the more clay-rich soils of the Tallahassee Red Hills (latitude 30.46277, longitude −84.26535) presented much steeper gradients than the sandy soils of Ant Heaven and the flatwoods, with the array in a wooded area reaching 4% CO_2_ at 175 cm, and the one in lawn reaching 1.6%. For comparison, at the same depth in the sandy soils of Ant Heaven, CO_2_ concentrations ranged from 0.25 to 0.8%. Only near the water table did the values exceed 1%. Gradients were somewhat steeper in the flatwoods, but here too, CO_2_ concentrations exceeded 1% mostly just over the water table.

## Discussion

The vertical structure of *P. badius* nests as well as the arrangement of ants and seeds within these nests makes it clear that the ants have information about depth below the surface. They do not appear to extract this information from the most apparent source, the soil CO_2_ gradient. Nest architecture and vertical sorting are similar even when the soil CO_2_ gradient is eliminated or reversed. The nature of the depth information is thus still unknown. It is possible that the ants arrange themselves socially by age, that is, only in reference to each other, and that the location of brood and nest structure are a consequence of this vertical social structure. This seems unlikely, as Tschinkel [1] found that single-age groups of workers build nests of similar shape, although different absolute size (old workers dig the largest). Perhaps work groups become socially organized in such a way as to produce the observed architecture. Moreover, callows arrange themselves without experience of the ground surface. Determining the mechanism through which ants construct their nests and sort themselves within it will undoubtedly remain a challenge for years to come.

Because soil CO_2_ is produced from organic matter by oxidation, CO_2_ gradients are really dual gradients– increased CO_2_ with depth, and a complementary decrease of O_2_ such that the two partial pressures sum to 21% of atmospheric. At low CO_2_ concentrations, the decrease in O_2_ is probably of little importance to organisms. For example, an increase of soil CO_2_ to 0.5% represents an approximately 15-fold increase from normal atmospheric, but the accompanying O_2_ decrease from 21% to 20.5% is only a 2% change. On the other hand, organisms deep in the soil, especially near the water table, may face CO_2_ concentrations of 5–10% accompanied by O_2_ concentrations of 16 to 11%. Such large differences in soil gases must be of importance to many soil-dwelling creatures. Some, such as certain species of earthworms, seem able to tolerate low O_2_ and very high CO_2_ concentrations [38, cited in 39], but the effects of soil gases on the physiology of soil dwelling creatures has rarely been investigated. Although the presence of carbon dioxide in ant nests has long been established [40], [41], studies of its possible effects are few. Leafcutter ants (*Atta* spp.) normally operate in CO_2_ concentrations of 4–5% or more [42], but the physiological or behavioral effect this has on either ants or fungus has been poorly explored.

The dangers of appealing hypotheses based on strong correlations are made abundantly clear by the present study, as is the desirability of publishing negative results. In light of the absence of a role of CO_2_ in the nest architecture and social arrangements of *P. badius*, it is interesting that experimental tests of the gaseous gradient hypothesis [33] in the ant *Leptothorax unifasciatus* [43] found no evidence that the ants used such gradients for sorting brood [34]. In any case, because Cox and Blanchard [33] assumed the CO_2_ to have been generated by the ants, their model applies only to ants nesting above ground between stones. In soil, any ant-generated CO_2_ would be swamped by that generated by microbes and roots, and would not produce a detectable signal. In soils as porous and organic-poor as those of Ant Heaven, the ants in colonies of *P. badius* do not detectably elevate nest CO_2_ above that of the ambient soil around them. Even fungus gardening ants with large colonies (*Atta* spp.) in whose nests the fungus produces a great deal of CO_2_ are not exceptions– although tunnels are passively ventilated by architectural means, CO_2_ and O_2_ concentrations in the fungus chambers themselves closely reflect that in the surrounding soil [42].

Hangartner [17] found that *S. geminata* workers dig less as the CO_2_ concentration increases, exactly what would be expected if the CO_2_ gradient served as a template for nest architecture. Moreover, Wilson [44] found that workers of *S. invicta* settled down in response to CO_2_, a result that might have supported the vertical distribution of harvester ants by age. My experiments seem to have eliminated the soil CO_2_ gradient as a template for either phenomenon in *P. badius*. Of course, it is still possible that species of *Solenopsis* use CO_2_ as a template while *P. badius* do not. The cues that guide the architecture and arrangement of *P. badius* are currently still a mystery.
